# Development of the Knee Quality of Life (KQoL-26) 26-item questionnaire: data quality, reliability, validity and responsiveness

**DOI:** 10.1186/1477-7525-6-48

**Published:** 2008-07-10

**Authors:** Andrew M Garratt, Stephen Brealey, Michael Robling, Chris Atwell, Ian Russell, William Gillespie, David King, the DAMASK Trial Team

**Affiliations:** 1 National Resource Centre for Rehabilitation in Rheumatology, Pb 23, Vinderen, 0319 Oslo, Norway; 2 Department of Health Sciences, York Trials Unit, Seebohm Rowntree Building, University of York, Heslington, York YO10 5DD, UK; 3 Department of Primary Care and Public Health, Cardiff University, Neuadd Meirionydd, Heath Park, Cardiff, CF14 4YS, UK; 4 Institute of Medical and Social Care Research, Brigantia Building, Penrallt Road, University of Wales Bangor, Gwynedd, LL57 2AS, UK; 5 Hull York Medical School, University of York, York, YO10 5DD, UK; 6 X-ray Department, York Hospital, Wigginton Road, York, YO31 8HE, UK

## Abstract

**Background:**

This article describes the development and validation of a self-reported questionnaire, the KQoL-26, that is based on the views of patients with a suspected ligamentous or meniscal injury of the knee that assesses the impact of their knee problem on the quality of their lives.

**Methods:**

Patient interviews and focus groups were used to derive questionnaire content. The instrument was assessed for data quality, reliability, validity, and responsiveness using data from a randomised trial and patient survey about general practitioners' use of Magnetic Resonance Imaging for patients with a suspected ligamentous or meniscal injury.

**Results:**

Interview and focus group data produced a 40-item questionnaire designed for self-completion. 559 trial patients and 323 survey patients responded to the questionnaire. Following principal components analysis and Rasch analysis, 26 items were found to contribute to three scales of knee-related quality of life: physical functioning, activity limitations, and emotional functioning. Item-total correlations ranged from 0.60–0.82. Cronbach's alpha and test retest reliability estimates were 0.91–0.94 and 0.80–0.93 respectively. Hypothesised correlations with the Lysholm Knee Scale, EQ-5D, SF-36 and knee symptom questions were evidence for construct validity. The instrument produced highly significant change scores for 65 trial patients indicating that their knee was a little or somewhat better at six months. The new instrument had higher effect sizes (range 0.86–1.13) and responsiveness statistics (range 1.50–2.13) than the EQ-5D and SF-36.

**Conclusion:**

The KQoL-26 has good evidence for internal reliability, test-retest reliability, validity and responsiveness, and is recommended for use in randomised trials and other evaluative studies of patients with a suspected ligamentous or meniscal injury.

## Background

The recognition that randomised trials and similar forms of evaluative study should include patients' views about outcome has contributed to a huge growth in the development and testing of instruments that measure aspects of health and quality of life from the perspective of the patient [1]. The majority have been developed for specific patient populations [1], including patients with knee problems [2]. Several generic instruments that are suitable for application across different patient populations have also been evaluated in patients with knee problems [2], including the EQ-5D [3] and SF-36 [3-9], the two most widely evaluated measures of health status [1].

In selecting an instrument for a specific population or type of knee problem, it is important that consideration is given to both questionnaire content and the population in which it has been evaluated [10]. For an instrument to be considered appropriate for assessing the health outcomes of patients with a specific knee problem supporting evidence including reliability, validity and responsiveness must be available. Furthermore, if an instrument is to have content validity as a measure of health outcome that is relevant to patients, then its content should be based on the views of patients [10]. It follows that an instrument for patients' with knee problems must be based on an assessment of the impact of the condition on patient quality of life.

Sixteen instruments that are specific to knee problems with evidence for reliability and validity were identified by a recent systematic review [2]. Such a large number can confuse clinicians and researchers wishing to select an instrument for application in randomised trials and clinical practice. The lack of standardisation in the choice of instruments limits the generalisability of results. Furthermore, of the five instruments included in the review that were based on the views of patients in relation to instrument development and the content of individual questions, there was no instrument specific to ligamentous or meniscal injury of the knee that also had adequate evidence for reliability and validity. An instrument specific to ligamentous or meniscal injury of the knee that is based on the views of patients will have greater content validity and hence is more likely to be responsive to changes in quality of life that are important to patients. These measurement properties are a prerequisite for a patient-reported instrument that is to be used in randomised trials and other forms of evaluative research.

This article describes the development of a knee-specific quality of life instrument based on the views of patients that has been used in a randomised trial and survey evaluating whether general practitioners (GPs) should have access to Magnetic Resonance Imaging (MRI) for patients with a suspected ligamentous or meniscal injury. The instrument was developed following in-depth interviews with patients and was assessed against criteria necessary for a self-reported instrument that will be used as an outcome measure within randomised trials and other forms of evaluative research including data quality, reliability, validity and responsiveness to change [10].

## Methods

### Instrument development

In-depth interviews were conducted with a pre-determined sample size of 35 patients to elicit how their knee injury affects their lives. Purposive sampling was used to select a stratified sample in relation to age, sex, severity, stage of management and condition – meniscal or ligamentous injuries. Interviews were conducted in two centres, Cardiff and York. They were audio-recorded and transcribed. Thematic analysis of the transcripts was undertaken by two researchers independently.

The resulting items were reviewed by the trial management group and piloted through a postal survey of 80 patients recruited from orthopaedic and physiotherapy departments at York Hospitals NHS Trust. Questionnaires were assessed for data quality including missing data and response frequencies. The questionnaire also included a question asking patients if they would be willing to attend an interview to discuss the questionnaire in more detail. These cognitive debriefing interviews conducted with twelve patients, were designed to assess whether they had any difficulties with particular items and if there were important areas not covered by the questionnaire.

Focus groups held with clinicians and patients in Cardiff and York were designed to assess content and face validity; the extent to which the items address the intended subject matter and whether relevant aspects of health and quality of life are adequately covered. The clinician focus groups included two GPs and three physiotherapists (Cardiff) and a GP, physiotherapist and consultant radiologist (York). The patient focus groups included three (Cardiff) and six patients (York). All focus groups followed a semi-structured topic guide and patients completed the questionnaire.

### Data collection

The instrument was evaluated concurrently within the randomised trial designed to evaluate whether GPs should have access to MRI for patients with a suspected ligamentous or meniscal injury. Patients completed a questionnaire at trial enrolment that also included the EQ-5D [11], SF-36 [12] and questions relating to knee symptoms. Patients who presented in general practice were eligible for inclusion in this trial if aged between 18 and 55 years inclusive and the GP was considering referral to an orthopaedic specialist for suspected meniscal or ligamentous injuries. The main exclusion criteria were that the patient needed urgent orthopaedic referral or had non-traumatic arthropathy, chronic instability of the knee, a previous MRI examination within the same episode of care, or previous surgical intervention (excluding diagnostic arthroscopy) on the same knee. These eligibility criteria were designed to help ensure a valid sample of patients were included in the trial. For example, the upper age limit of 55 years inclusive was used as this is when the onset of osteoarthritis occurs [13]. The injuries consistently referred to are those of the menisci, and cruciate or collateral ligaments [14,15] so these are the type of patients GPs were asked to recruit. GP's also attended a two-hour education seminar that included the clinical assessment and management of patients with knee problems [16].

Questionnaires were also sent to patients invited to take part in a postal survey in Cardiff which comprised two cohorts: all recent GP referrals to the University Hospital of Wales and Llandough Hospital for MRI or orthopaedic consultation; and patients listed for an arthroscopy of the knee. These questionnaires included the Lysholm Knee Score [17], a widely used clinically-derived instrument for assessing ligament injuries of the knee. Postal reminders were sent at two and four weeks, and patients were contacted by telephone at six weeks.

### Statistical analysis

Individual items were assessed for missing data. Principal component analysis (PCA) was used to identify interrelated variables or components of quality of life within the questionnaire items [18]. Items with poor component loadings were considered for removal. Items were further assessed for their contribution to the identified components with Rasch analysis [19], a form of item response theory (IRT) using the WINSTEPS programme [20]. IRT assumes that a measurement construct such as knee-related quality of life can be represented by an ordered continuum that ranges from the minimum to maximum level of functioning. IRT models produce a hierarchy in which items are positioned according to the proportion of patients who report a limitation, and patients are positioned according to the total number of items in which they report a limitation [21]. Two mean square fit statistics assess the extent to which unpredicted responses to an item are given by patients whose position in the hierarchy as determined by their knee-related quality of life, is either close to the items position (Infit statistic) or far from the items position (Outfit statistic) in the hierarchy of items. It is generally recommended that the two fit statistics range from 0.7–1.3 [21,22].

The instrument was assessed for internal consistency reliability using item-total correlation and Cronbach's alpha [10]. Test-retest reliability was assessed through a second questionnaire mailed to a random sample of every second patient taking part in the GP referral cohort of the Cardiff survey at two weeks. Patients reporting no change on the knee-specific transition question "Compared with 2 weeks ago, how is your knee now?" were included in the test-retest analysis [10,23]. To be acceptable for use in groups of patients the estimates of reliability should exceed 0.70 [10,24].

The validity of the new instrument was assessed through comparisons with self-reported health status instruments and questions about knee symptoms. It was hypothesised that the new instrument would have a higher level of correlation of around 0.7 with the Lysholm Knee Score than the two generic instruments the EQ-5D and SF-36. Moderate levels of correlation of 0.5 were expected with the EQ-5D scores. Higher levels of correlation were expected for the EQ-5D items relating to mobility and pain than for self-care or anxiety-depression. Similarly, higher correlations in the range 0.5–0.7 were expected for the SF-36 scales of physical function, role-physical and bodily pain than for the scales of general health, vitality and mental health. Low to moderate levels of correlation above 0.4 were expected with patient responses to questions about number of days off work or normal activities in the past four weeks. It was hypothesised that instrument scores would reflect the different categories of symptoms in the form of a linear association, the highest and lowest KQoL-26 scores being for patients having symptoms none of the time and all of the time respectively. It was hypothesised that patients who had or were considering changing job because of their knee would have lower scores than their counterparts.

Responsiveness was assessed on return of a follow-up questionnaire at six months for the trial patients. This questionnaire included a knee-specific health transition question "Compared with six months ago, how is your knee now?" which uses a 15 point scale from "a very great deal worse" to "a very great deal better". An improvement of a little or somewhat better has been defined as important [23]. Responsiveness was compared using effect sizes and responsiveness statistic. The former is equal to the mean change in score divided by the standard deviation of the baseline scores [10]. The latter is equal to the mean change in scores divided by the standard deviation of score differences in stable patients [25]. The larger the responsiveness statistic, the smaller the sample size needed for purposes of evaluation. The denominator was the standard deviation of the score changes for those patients in the test-retest analysis.

### Ethics

The study was designed to comply with the Declaration of Helsinki as adopted by the World Medical Association. Northern and Yorkshire Multi-Centre Research Ethics Committee reviewed and approved the study (reference number MREC/1/3/59).

## Results

### Instrument development

Analysis of the interview transcripts produced a 38-item instrument which was scaled with three types of five-point adjectival scales of "totally limited/unable to do" to "not limited at all", "all of the time" to "none of the time" and "extremely" to "not at all". The instrument was piloted using a postal survey and 47 (58.8%) were returned completed. Twelve questionnaires were also completed by patients attending the focus groups. With regards to missing data, when piloting the questionnaire 52 (88.1%) patients completed all items. The focus groups and follow-up interviews confirmed the content and face validity of the questionnaire following the addition of two items about financial concerns and feelings of getting old relating to the knee problem.

### Data collection

The 559 patients who took part in the trial completed the 40-item questionnaire. Of the 547 patients invited to take part in the Cardiff survey, 323 (59.0%) returned a completed questionnaire. For the trial and survey patients the mean ages were 39.67 (sd = 10.23) and 47.02 (sd = 14.29) and the number of females was 204 (36.6%) and 146 (43.5%) respectively (Table 1).

**Table 1:** Patient characteristics

	Trial entry assessment^a^		
Characteristics	MRI referral (n = 279)	Orthopaedic referral (n = 274)	Survey patients (n = 323)
Mean age, years (SD)	40.1 (9.9)	39.1 (10.5)	47.0 (14.3)
Sex, n (%)			
Male	185 (66)	164 (69)	177 (56)
Female	94 (34)	109 (40)	146 (44)
Diagnostic category, n (%)			
Meniscal injury	224 (80)	210 (77)	
Ligamentous injury	87 (31)	82 (30)	
Other diagnosis	5 (2)	9 (3)	
No diagnosis	0 (0)	0 (0)	

^a^Information was not available for six patients.

### Statistical analysis

The majority of items have four or fewer missing responses in total, the mean number of missing responses being 0.12 (sd = 0.67) with a range of 0 to 9 (2.3%) patients that did not respond to a particular item. All forty items were completed by 751 (85.1%) patients. The largest number of missing responses was eleven items for one patient which is just over one quarter of the items. Across the 0–4 response scale, means ranged from 1.05 to 3.47 for the items "avoiding turning, twisting or sideways movements" and "crossing the road" respectively. The former item had the highest floor effect of 35.4%. The item "staying seated for 15 minutes" had the highest ceiling effect of 62.0%. The spread of the item means indicates that items are measuring different degrees of knee-related quality of life.

Principal component analysis gave five components with eigenvalues above 1.0 explaining 64.1% of the total variation between patients. The great majority of the items were making a strong contribution to the first component of the unrotated solution. Rotation served to identify clinically recognisable aspects of knee-related quality of life; the first, second and fourth components comprising items relating to physical functioning, activity limitations and emotional functioning respectively. The third and fifth components included six items relating to getting into or out of a lowered position and two items relating to bending and kneeling down respectively. The majority of these items and items within the physical functioning component had higher loadings within the first factor of the unrotated solution and the majority had loadings over 0.4 within the first component of the rotated solution. They were therefore assessed for their contribution to the hypothesised scale of physical functioning. As shown in Table 2, all items have component loadings above 0.5, the great majority above 0.6.

**Table 2:** Principal component analysis, item-total correlation, logit measure and fit statistics (n = 559)

					Fit statistics	
Scale/item		Component loadings	Item-total correlation	Logit measure^a ^(error)	Infit MNSQ	Outfit MNSQ
*Physical functioning*						
1	Lifting heavy objects	.69	.68	-0.15 (0.08)	1.00	1.05
2	Running	.62	.60	2.69 (0.07)	1.18	1.17
3	Walking on uneven ground	.67	.68	0.75 (0.07)	0.87	0.90
4	Walking five miles	.70	.69	1.95 (0.07)	1.14	1.10
5	Walking one mile	.71	.74	0.29 (0.07)	1.11	1.04
6^b^	Walking 100 yards	.68	.72	-1.62 (0.11)	0.87	0.81
7	Crossing the road	.67	.68	-2.25 (0.14)	0.92	0.81
8^b^	Bending down	.53	.55	0.62 (0.07)	1.36	1.37
9	Kneeling down	.63	.63	2.25 (0.07)	1.00	0.98
10	Standing for one hour	.71	.72	0.52 (0.07)	1.09	1.07
11	Standing for five minutes	.66	.66	-1.56 (0.12)	0.94	0.85
12	Going up several flights of stairs	.77	.79	1.09 (0.07)	0.71	0.72
13	Going up one flight of stairs	.75	.78	-0.43 (0.08)	0.71	0.73
14	Going down several flights of stairs	.75	.75	0.88 (0.07)	0.78	0.79
15	Going down one flight of stairs	.73	.75	-0.36 (0.08)	0.74	0.73
16	Getting into a sitting position	.67	.68	-1.24 (0.10)	1.02	0.97
17^b^	Staying seated for two hours	.55	.53	0.01 (0.08)	1.55	1.50
18^b^	Staying seated for 15 minutes	.58	.59	-1.49 (0.12)	1.11	0.94
19^b^	Getting up from a sitting position	.65	.67	-0.35 (0.08)	0.99	1.00
20^b^	Getting in or out of the bath or shower	.71	.73	-0.55 (0.08)	0.89	0.89
21	Getting in or out of bed	.67	.68	-1.06 (0.09)	0.92	0.96
*Activity limitations*						
22	Knee slowing you down	.62	.76	0.69 (0.07)	0.69	0.74
23^b^	Avoid turning/twisting/sideways moves	.57	.56	1.66 (0.07)	1.23	1.40
24	Interference with travel	.56	.71	-0.88 (0.08)	1.29	1.24
25^b^	Interference with sleep	.50	.64	-0.72 (0.07)	1.38	1.41
26	Interference with work	.59	.78	0.31 (0.07)	0.79	0.80
27^b^	Interference with hobbies, etc	.62	.51	1.52 (0.07)	1.30	1.27
28^b^	Interference with family life	.59	.80	-0.46 (0.07)	0.66	0.65
29	Interference with social activities	.56	.81	-1.11 (0.08)	0.80	0.77
30	Interference with doing errands	.53	.80	-1.02 (0.08)	0.83	0.80
*Emotional functioning*						
31	Time spent thinking about knee	.64	.71	1.34 (0.07)	0.84	0.94
32	Angry or annoyed	.73	.74	0.61 (0.07)	0.83	0.78
33	Downhearted and low	.78	.80	-0.23 (0.07)	0.82	0.80
34	Worried about knee worsening	.78	.73	0.97 (0.07)	0.90	0.85
35^b^	Embarrassed or self-conscious	.62	.68	-1.16 (0.08)	1.17	1.10
36	Bad temper or grumpy	.72	.76	-1.14 (0.08)	0.77	0.77
37	Frustrated	.69	.75	0.38 (0.07)	0.76	0.74
38^b^	Worry about future	.79	.73	0.64 (0.07)	0.90	0.94
39^b^	Worry about money	.60	.58	-1.03 (0.09)	1.62	1.58
40^b^	Think that you are getting old	.52	.39	-0.38 (0.08)	1.79	1.87

^a ^Logits of lower magnitude represent decreasing quality of life.

^b ^Item removed from final version.

Table 2 shows that for three scales the majority of items fit the Rasch model with Infit and Outfit statistics between 0.8 and 1.2, the great majority within the recommended range 0.7–1.3 [22]. This is further evidence that the instrument comprises three scales of knee-related quality of life. Within physical functioning, items 8 "bending down" and 17 "staying seated for two hours" have poor Infit and Outfit statistics which indicates that they do not fit the Rasch model very well and may not be sufficiently contributing to the construct of physical functioning. Similarly, three items within activity limitations and two items within emotional functioning have poor fit statistics. Item 22 is borderline.

For the three scales, all but one of the item-total correlations are above 0.40. Removal of items 6, 8, 17, 18, 19 and 20 from the physical functioning scale which includes those performing poorly in the Rasch analysis, gave final item-total correlations of 0.60–0.81 and a Cronbach's alpha of 0.94. Removing four items from the activity limitations scale including those performing poorly in the Rasch analysis, gave final items-total correlations of 0.70–0.82 and an alpha of 0.91. Removing four items from the emotional functioning scale including those performing poorly in the Rasch analysis, gave final item-total correlations of 0.74–0.81 and an alpha of 0.91.

Of the 109 randomly selected patients from the GP referral cohort of the Cardiff survey, 86 (78.9%) completed a two-week retest questionnaire. The intra-class correlations for the two sets of scores for the 27 patients stating that there was no change in their knee were 0.93, 0.87 and 0.80 for the physical functioning, activity limitations and emotional functioning scales respectively. These reliability estimates meet the criteria necessary for group level comparisons [10,26].

The score distributions for the new instrument now referred to as the KQoL-26, are computed by summing the items where half or more are completed for the relevant scale and transforming to a 0–100 scale. The scores were approximately normal on a scale of 0–100 where 100 is the best possible knee-related quality of life. Means scores were 59.1 (sd = 18.8), 51.05 (sd = 25.2) and 40.4 (sd = 21.4) for the physical functioning, activity limitations and emotional functioning scales respectively. Therefore the instrument has the potential to measure differences in knee-related quality of life in cross-sectional or longitudinal studies.

The results of the tests of validity are shown in Tables 3 and 4. The correlations between KQoL-26 scores, the Lysholm, EQ-5D, SF-36 and knee-related questions are shown in Table 3. As hypothesised the highest correlations are with the Lysholm Knee Score. This is higher than that hypothesised which may reflect the high level of reliability of the new instrument [10,26]. As hypothesised, in general the KQoL-26 had the highest correlations with aspects of the EQ-5D and SF-36 relating to physical health. The KQoL-26 physical functioning and activity limitations had the largest correlations with the EQ-5D mobility and pain items. As expected the emotional functioning scale had the largest correlation with the EQ-5D anxiety and depression item. The KQoL scales had a similar moderate level of correlation with EQ-5D index scores. The KQoL-26 physical functioning and activity limitations scales had the largest correlations with the SF-36 scales of physical functioning, social functioning, bodily pain and role-physical. The emotional functioning scale had the largest correlations with social functioning and role-emotional. This KQoL scale also had the highest correlation with the SF-36 mental component summary scores, the other two scales having higher correlations with the physical component summary scores. The correlations with the number of days off work and on which normal activity was prevented because of the knee were of a low to moderate level. As expected, the activity limitations scale scores had the highest level of correlations with responses to these two questions.

**Table 3:** Correlation between KQoL-26 scores, EQ-5D, Lysholm Knee Score, SF-36 and days affected by knee problem (n = 559)

	KQoL-26 scales		
Variable	Physical functioning	Activity limitations	Emotional functioning
EQ-5D score	.54	.49	.50
Mobility	.52	.49	.35
Self-care	.35	.31	.21
Usual activities	.31	.31	.31
Pain/discomfort	.43	.38	.40
Anxiety/depression	.32	.34	.45
			
Lysholm Knee Score^a^	.76	.76	.58
			
SF-36:			
Physical functioning	.75	.65	.50
Role physical	.54	.61	.55
Bodily pain	.54	.62	.55
General health	.25	.22	.21
Vitality	.43	.44	.48
Social functioning	.57	.66	.60
Role emotional	.47	.52	.56
Mental health	.41	.40	.51
			
Physical Component Summary	.64	.63	.47
Mental Component Summary	.39	.44	.55
			
Days off work due to knee	.25	.38	.28
Days normal activity prevented	.38	.46	.43

^a ^Cardiff survey data (n = 323)

All correlations are statistically significant (p < 0.01)

**Table 4:** Mean (standard deviation) KQoL-26 scores^a ^by responses to knee-related questions

Variable	Physical functioning		Activity limitations		Emotional functioning	
	n	mean (sd)	n	mean (sd)	n	mean (sd)
*Knee pain present during day:*						
None of the time	7	71.52 (19.44)	7	80.00 (23.98)	7	73.71 (21.27)
Some of the time	305	65.91 (16.93)	306	60.74 (21.97)	306	47.55 (19.78)
All of the time	237	49.93 (17.50)	235	37.43 (22.69)	237	30.08 (18.65)
*Knee pain present at night:*						
None of the time	64	74.51 (14.47)	64	73.75 (18.90)	64	54.16 (22.13)
Some of the time	345	60.47 (17.54)	344	53.09 (23.31)	346	42.62 (20.03)
All of the time	144	48.92 (18.52)	141	35.57 (22.75)	141	28.55 (18.87)
*Knee pain worse on movement:*						
None of the time	12	72.15 (18.63)	12	69.72 (25.27)	12	65.67 (23.85)
Some of the time	264	65.62 (17.97)	263	59.84 (22.56)	265	46.02 (20.89)
All of the time	273	52.25 (17.32)	273	41.65 (24.30)	273	33.70 (19.48)
*Knee stiff in morning:*						
None of the time	110	69.18 (17.32)	110	64.92 (23.01)	110	49.08 (21.64)
Some of the time	237	60.45 (18.07)	237	52.63 (23.84)	237	41.59 (21.03)
All of the time	201	52.21 (18.15)	201	41.62 (24.22)	201	33.79 (19.81)
*Duration of morning stiffness:*						
Less than 1 hour	63	61.23 (18.20)	63	53.40 (23.55)	63	42.31 (20.46)
1 to 2 hours	112	50.52 (17.55)	112	39.65 (23.85)	112	32.84 (19.09)
Over 2 hours	268	49.85 (18.11)	268	38.49 (25.07)	268	30.43 (21.06)
*Knee given way when walking:*						
Never	166	65.36 (18.76)	166	59.80 (24.13)	166	47.27 (20.83)
Over three months ago	42	67.74 (18.77)	42	57.86 (25.47)	42	45.43 (22.40)
One to three months ago	48	61.97 (18.41)	48	54.90 (24.70)	48	39.58 (21.38)
In the last month	293	53.80 (17.57)	293	44.27 (24.09)	293	35.77 (20.61)
*Painful inability to straighten knee:*						
Never	135	66.61 (16.75)	135	59.01 (24.71)	135	47.68 (22.24)
Over three months ago	39	66.54 (17.39)	39	59.49 (24.70)	39	45.95 (21.22)
One to three months ago	48	64.55 (17.98)	48	46.38 (23.60)	48	41.46 (22.32)
In the last month	324	54.33 (18.64)	324	45.90 (24.52)	324	36.59 (20.09)
*Presence of knee swelling:*						
Never	118	66.41 (17.83)	117	61.85 (24.14)	118	47.97 (21.70)
Over three months ago	35	72.95 (15.87)	35	68.71 (20.52)	35	54.51 (21.17)
One to three months ago	58	60.83 (17.61)	58	51.90 (26.40)	58	38.79 (20.79)
In the last month	333	55.06 (18.09)	333	45.32 (23.64)	334	36.55 (20.08)
*Changed job due to knee:*						
No	506	58.79 (16.17)NS	505	51.02 (24.93)NS	507	40.47 (21.28)NS
Yes	18	57.13 (18.68)	18	41.67 (25.14)	18	36.67 (20.04)
*Considering job change due to knee:*						
No	486	59.54 (18.92)NS	485	51.91 (24.85)	487	41.41 (20.93)
Yes	27	53.55 (18.41)	27	36.11 (20.95)	27	28.15 (19.80)

^a ^KQoL-26 scores range from 0 to 100;0 is the worst possible and 100 the best possible knee-related quality of life

All results are statistically significant (p < 0.01) unless indicated; NS not significant

Table 4 shows the results of the tests of validity in comparison to knee symptoms and change of job. The results are statistically significant for 26 of the 30 tests. The sample sizes are relatively small for those patients who have changed or are considering changing their job because of their knee, however three of the results are statistically significant, the largest differences being found for the activity limitations scale.

Of the 559 trial patients sent a six month questionnaire, 472 (84.4%) responded. Of these, 65 indicated that they had undergone an improvement of "a little better or somewhat better" on the knee-related health transition question. Table 5 shows the score changes, effect sizes and responsiveness index for these patients. With the exception of four SF-36 scale scores, all the instruments show a significant score improvement at six months. The KQoL-26 shows an improvement of 16.41, 23.38 and 23.46 points on scales of physical functioning, emotional functioning and activity limitations respectively. The KQoL-26 has the three highest effect sizes and responsiveness statistics.

**Table 5:** Mean (standard deviation) scores and responsiveness index for trial patients (n = 65) who have undergone a knee-related health improvement of "a little better or somewhat better" at six months

Variable	Baseline	Six months	Change	Effect size^a^	Responsiveness index^b^
KQoL-26:					
Physical functioning	57.08 (18.99)	73.49 (15.44)	16.41 (16.95)**	0.86	2.13
Activity limitations	51.15 (25.86)	74.62 (16.96)	23.46 (20.19)**	0.91	1.86
Emotional functioning	40.92 (20.73)	64.31 (19.84)	23.38 (15.46)**	1.13	1.50
					
EQ-5D	0.60 (0.26)	0.72 (0.13)	0.12 (0.24)**	0.46	0.51
					
SF-36:					
Physical functioning	54.77 (25.87)	69.89 (19.54)	15.12 (21.33)**	0.58	1.12
Role physical	54.90 (32.09)	69.33 (23.38)	14.42 (26.86)**	0.44	0.86
Bodily pain	46.17 (26.67)	63.40 (19.62)	17.23 (21.71)**	0.65	0.78
General health	69.57 (18.08)	66.08 (19.97)	-3.49 (14.60)	-0.19	0.23
Vitality	52.40 (18.84)	53.94 (18.90)	1.54 (18.05)	0.08	0.08
Social functioning	66.54 (27.07)	78.08 (20.61)	11.54 (24.44)**	0.42	0.93
Role emotional	77.18 (31.12)	85.77 (19.02)	8.59 (27.24)*	0.28	0.59
Mental health	70.38 (19.21)	70.92 (16.81)	0.54 (14.42)	0.03	0.03
					
Physical Summary	38.90 (10.52)	44.63 (8.74)	5.73 (9.41)**	0.55	1.06
Mental Summary	48.74 (12.25)	49.32 (10.17)	0.58 (9.71)	0.05	0.09

^a ^ES = Mean change in score divided by the standard deviation of baseline score

^b ^RI = Mean change in score divided by the standard deviation of score changes in stable subjects

Asterisks denote statistical significance: *p < 0.05; **p < 0.01

## Discussion

There are a large number of knee-specific instruments for measuring the health status and outcomes of patients with knee problems. Few have been adequately evaluated for the measurement properties deemed necessary for self-reported measures of health and quality of life [2]. Only three of the five instruments that incorporate the views of patients have been adequately evaluated for reliability and validity. Of these, the Knee Pain Scale [27] and Oxford Knee Score [28] were developed in patients with osteoarthritis and there is limited empirical evidence for the internal construct validity, including the results of factor and principal component analysis, of the scales of the Knee Injury and Osteoarthritis Outcome Score [2,29]. The WOMAC is another instrument that has had wide application, but for patients with osteoarthritis and was not developed solely for knee problems [30]. Therefore the instrument has less content validity as a measure of the effect of a patient's knee problem on their quality of life and may also be less responsive than an instrument that is specific to the knee [10].

The KQoL-26 is a self-reported questionnaire for assessing knee-related quality of life that is based on the views of patients and satisfies the requirements of a patient-reported outcome measure that is suitable for randomised trials [10]. The instrument has undergone a more extensive evaluation than the great majority of instruments described in a recent systematic review [2]. Based on patient interviews, focus groups, piloting and follow-up interviews, instrument development ensured that the views of patients with knee problems formed the basis of item construction. Therefore the KQoL-26 has high content validity as a measure of knee-related quality of life. The instrument was then evaluated for reliability, construct validity and responsiveness to change in a large sample of patients with a suspected ligamentous or meniscal injury. The result is an instrument that has the measurement characteristics necessary for a patient-reported outcome measure for use in evaluation [10].

The response rate to the Cardiff survey of 59.0% was considerably lower than the trial where patients had a stronger incentive to return a completed questionnaire. The KQoL-26 takes patients around five minutes to complete which is acceptable for an instrument that is to be used alongside other instruments within a questionnaire booklet for assessing health outcomes. The low levels of missing data for the 26 items are further evidence that the instrument is acceptable to patients as a self-reported postal questionnaire. Scale scores were computable for 557 (99.6%) of the 559 trial patients who returned a baseline questionnaire. The results of principal component analysis and Rasch analysis are strong evidence that the 26-items contribute to three knee-related quality of life scales: physical functioning, activity limitations and emotional functioning. The final item-total correlations ranged from 0.6 to 0.82, which are within widely accepted standards [10,24,26]. The estimates for internal and test-retest reliability are all above 0.80 which suggests that the instrument is suitable for use in applications involving groups of patients including randomised trials. Physical functioning, the most important scale for capturing variation between patients and the most responsive according to the responsiveness index, may be appropriate for use by individual patients, including clinical practice [10].

Twelve of the 16 knee-specific instruments included in a systematic review have been tested for test-retest reliability, the majority of which produced test-retest correlations over 0.7 [2]. The present study asked patients to complete a health transition question so that only those patients stating that there had been no change in their knee condition in the two weeks between administrations were included in the test-retest analysis. None of the test-retest studies reported in the review used transition questions but the great majority had shorter times between test and retest questionnaires of between 1 and 14 days to ensure that the patients' knee condition had not changed [2]. The reliability estimates for the KQoL-26 are acceptable and over 0.8, the lowest being for the emotional functioning scale which might be expected given the content of the knee transition question. Higher test-retest estimates might have been produced with three transition questions which directly related to the content of the three KQoL-26 scales.

The tests of responsiveness also followed the application of a criterion based on a health transition question. Patients were selected who stated that they had undergone an improvement of a little or somewhat better on a widely used transition question [23]. In contrast to tests of responsiveness of following treatment previously used to assess knee-specific instruments [2], such a criterion has the advantage that it is patient-reported and hence is designed to determine the level of change that is of importance to them. Using the criterion of treatment of known efficacy may show which instrument is the most responsive in relation to existing interventions, but the most common application of patient-reported outcome measures is in the evaluation of new interventions including the randomised comparison with existing interventions which will produce more marginal gains in terms of clinical effectiveness. Smaller levels of change or differences in patient outcomes between interventions are of greater relevance and hence it is important that instruments are responsive to what patients' perceive to be small levels of change.

The results of comparisons with other instruments and knee-related questions are evidence for the validity of the KQoL-26. The high level of correlation with the Lysholm Knee Score and moderate to high levels of correlation with EQ-5D and SF-36 scores are evidence that the instrument is measuring the effects of patient knee problems across the different aspects of health and quality of life assessed by these instruments. In particular, the highest correlations with these instruments were found for the KQoL-26 scales that we would expect to have the greatest association. For example, of the three KQoL-26 scales, physical functioning had highest correlations with the EQ-5D scores for mobility and SF-36 physical functioning scores and physical component summary scale. Activity limitations had the highest correlations with the SF-36 social functioning scores. This scale also produced the highest correlations with the number of days that work and normal activity was prevented. Emotional functioning had the highest correlations with EQ-5D scores for anxiety/depression and mental component summary scores. This scale also had the highest correlation of all with the SF-36 scale of role-emotional.

Nine existing knee instruments have been correlated with the SF-36 for purposes of assessing validity [2]. The range of the correlations found between the KQoL-26 and the SF-36 scales was generally higher than that found for existing instruments. In common with the majority of these instruments, the highest correlations were found between the KQoL-26 and the SF-36 scale of physical functioning. The Edinburgh Knee Function Scale [31], Knee Outcome Survey Activities of Daily Living Scale [32] and Oxford Knee Score [28] are the only instruments that based on the evidence included in this review, had correlations over 0.7 with the SF-36 physical functioning scale. Only the Oxford Knee Score had a larger correlation of 0.78 which was in a postoperative group; correlations for the pre-operative group were more modest. In contrast to the KQoL-26, none of the review instruments had correlations over 0.5 with the mental health and role-emotional scales of the SF-36. This shows the importance of the KQoL-26 emotional functioning scale which had the largest correlations with these two SF-36 scales. Moreover, none of the review instruments had such consistently high correlations with the SF-36 scales of social functioning and role-physical as the KQoL-26 scale of activity limitations. This is evidence that the KQoL-26 has evidence for validity that exceeds that for existing knee instruments.

KQoL-26 scores were also significantly related to knee symptoms, activity limitations and work limitations. Large differences were found for the emotional functioning scale, an aspect of quality of life not covered by existing knee-specific instruments [2]. This is further evidence that the new instrument is assessing the broad impact on patient quality of life.

Finally, the three KQoL-26 scales produced highly significant score changes in patients who had what is recognised to be an important improvement on a self-reported knee-related health transition question at six months [24]. The three scales scores were more responsive than the EQ-5D and SF-36 scales, particularly with respect to emotional functioning. Together with the evidence for the reliability and validity of the instrument, this further underpins the use of the KQoL-26 as a primary outcome measure within randomised trials.

## Conclusion

The KQoL-26 has good evidence for data quality, reliability, validity and responsiveness to important changes in health. The KQoL-26 is recommended as an instrument that assesses aspects of health and quality of life that are important to patients with ligamentous or meniscal injuries.

## Competing interests

The authors declare that they have no competing interests.

## Authors' contributions

AMG conceived and designed the study, conducted the analysis and drafted the manuscript. SB contributed to the design of the study, acquired ethical approval, collected data, and commented on the manuscript. MR contributed to the design of the study, acquired ethical approval, collected data, and commented on the manuscript. CA collected data, contributed to analysis of data from patient interviews and focus groups, and commented on the manuscript. IR contributed to the design of the study and commented on the manuscript. WG contributed to the design of the study and commented on the manuscript. DK contributed to the design of the study and commented on the manuscript. All authors read and approved the final manuscript.
